# Supplementary material for: The Denaturant- and Mutation-Induced Disassembly of Pseudomonas aeruginosa Hexameric Hfq Y55W Mutant
Source: Molecules. 2022 Jun 14;27(12):3821. doi: 10.3390/molecules27123821 (PMC9228748; doi:10.3390/molecules27123821)
Supplement: Supplementary file 1 [file molecules-27-03821-s001.zip › molecules-1714071-supplementary.pdf]

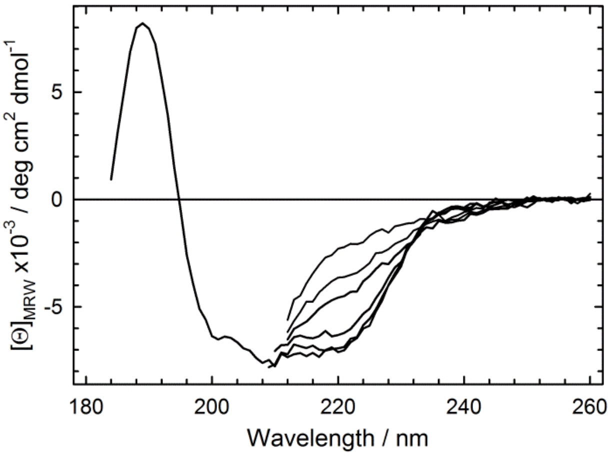


(**a**)

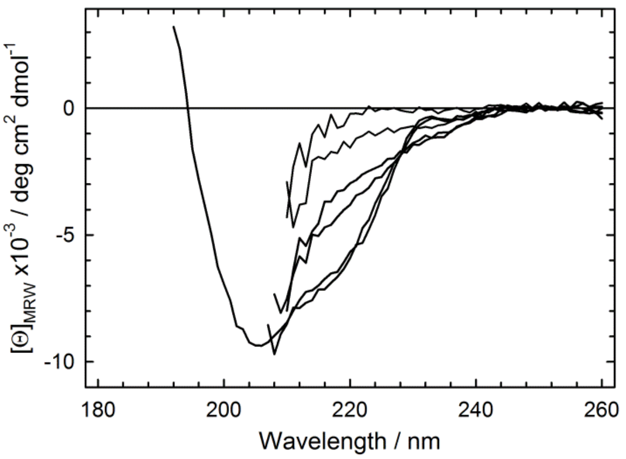


(**c**)

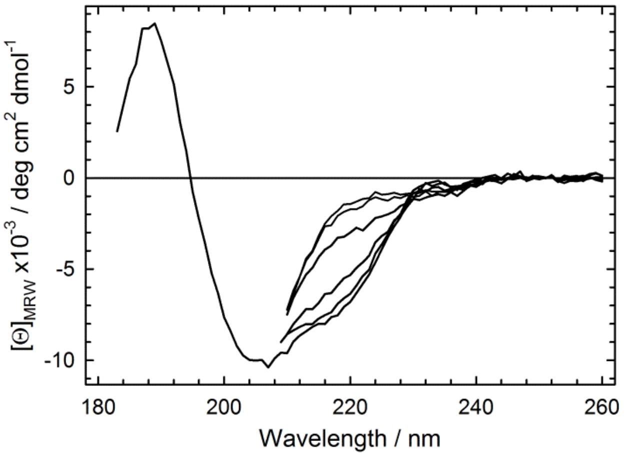


(**b**)

**Figure S1.** The selected far-UV CD spectra of Hfq variants for the crucial points of GuHCl-induced unfolding of the proteins: (**a**) WT Hfq at the protein concentration 66 μM (monomer), GuHCl concentration from bottom to top: 0 M, 2.74 M, 3.18 M, 4.48 M, 4.68 M, 5.47 M; (**b**) Hfq Y55W mutant at the protein concentration 66 μM (monomer), GuHCl concentration from bottom to top: 0 M, 1.44 M, 2.21 M, 2.67 M, 4.04 M, 5.1 M; (**c**) Hfq Y55W mutant at the protein concentration 6 μM (monomer), GuHCl concentration from bottom to top: 0 M, 1.44 M, 2.21 M, 2.67 M, 4.04 M, 5.1 M.
